# MLP-CKD: a clinically informed deep learning framework for admission laboratory-based screening and risk stratification of uremia-associated advanced renal dysfunction

**DOI:** 10.3389/fpubh.2026.1858083

**Published:** 2026-06-09

**Authors:** Xueliang Chen, Hao Yang, Yunxia Huang, Wen Jin, Xiaofeng Zhu, Junchen Zhao, Xiaoyi Huang

**Affiliations:** 1 Department of Nephrology, Pujiang County People's Hospital, Jinhua, Zhejiang, China; 2 The First Affiliated Hospital of Zhejiang University School of Medicine, Hangzhou, China; 3 Department of Nephrology, Zhejiang University School of Medicine, Hangzhou, China

**Keywords:** admission laboratory screening, deep learning, feature interaction, multi-branch architecture, multilayer perceptron (MLP), routine laboratory parameters, uremia-associated advanced renal dysfunction

## Abstract

**Objective:**

To develop and validate a clinically informed deep learning framework for admission laboratory-based screening and risk stratification of uremia-associated advanced renal dysfunction.

**Methods:**

We propose MLP-CKD, a multi-branch feature interaction-enhanced multilayer perceptron that groups laboratory variables by clinical domain and incorporates missingness indicators to capture test-availability patterns. Adult ICU patients were extracted from MIMIC-IV, and laboratory measurements obtained within the first 24 h after ICU admission were summarized using mean, minimum, and maximum values. To prevent information leakage, only the first eligible ICU stay per patient was retained, and all model development was conducted using patient-level training, validation, and independent test splits. MLP-CKD was compared against conventional machine learning models, neural network ablation variants, and clinical rule-based baselines. Performance was assessed using discrimination, calibration, decision curve analysis, bootstrap-based 95% confidence intervals, and permutation importance.

**Results:**

On the independent patient-level test set, MLP-CKD achieved an AUC of 0.913 (95% CI: 0.907–0.919) and an AUPRC of 0.848 (95% CI: 0.838–0.859), representing the best discrimination among the primary models. Its recall and F1-score were 0.796 and 0.764, respectively. Ablation analysis showed that the Branch-only MLP achieved near-equivalent discrimination (AUC 0.913; paired-bootstrap *p* = 0.952), indicating that clinically informed feature grouping was the main performance driver, whereas the interaction module provided only incremental benefit. MLP-CKD outperformed XGBoost (Delta AUC = 0.010, *p* < 0.001), random forest (Delta AUC = 0.016, *p* < 0.001), and the eGFR rule-based baseline (Delta AUC = 0.047, *p* < 0.001). Feature-restricted analyses suggested that the model integrated complementary laboratory information beyond creatinine and BUN alone.

**Conclusion:**

MLP-CKD provides a clinically structured framework for admission laboratory-based screening and risk stratification of uremia-associated advanced renal dysfunction. The model achieved competitive discrimination and clinically plausible feature-importance patterns, with its main advantage arising from clinically informed feature grouping. Because this retrospective study used admission-window laboratory variables without a predefined future prediction horizon, MLP-CKD should be interpreted as a decision-support tool rather than as a standalone diagnostic system or a model for predicting disease onset.

## 1 Introduction

Uremia-associated advanced renal dysfunction represents a clinically severe stage of kidney disease and is associated with substantial morbidity, frequent hospitalization, and increased healthcare burden (1, 2). Throughout this manuscript, the term “uremia-associated advanced renal dysfunction” refers to an operational outcome label indicating advanced kidney dysfunction or kidney failure status, defined using documented end-stage renal disease (ESRD)-related International Classification of Diseases (ICD) codes, renal replacement therapy records, or laboratory-derived estimated glomerular filtration rate (eGFR) < 15 mL/min/1.73 m^2^ when available. This operational definition follows the clinical framework of the KDIGO guideline and is described in detail in the Methods section (1). We use this term consistently in place of “uremia,” “renal dysfunction,” or “ESRD,” which carry distinct clinical meanings.

In clinical practice, the identification of advanced renal dysfunction relies on biochemical evidence, diagnostic history, renal replacement therapy status, and physician assessment (1, 3). Serum creatinine and blood urea nitrogen (BUN) are central indicators of impaired renal filtration and nitrogenous waste retention, but advanced renal dysfunction is rarely reflected by a single laboratory marker alone. Electrolyte disturbance, metabolic acidosis, anemia, mineral metabolism abnormalities, and nutritional deterioration may also accompany advanced kidney dysfunction and provide complementary clinical information (1, 4). In busy admission settings, systematically integrating these heterogeneous laboratory signals may be challenging, creating a need for structured laboratory-based screening tools that support admission-time risk stratification using routinely collected data.

With the increasing availability of electronic health records (EHRs), large-scale de-identified clinical databases such as MIMIC-IV have provided an important foundation for reproducible clinical prediction research (5, 6). Structured laboratory data are particularly attractive for clinical screening because they are routinely collected, widely available, and relatively inexpensive compared with imaging or specialized testing. Machine learning methods have been increasingly used in healthcare for disease classification, risk stratification, and clinical decision support (7–9). For structured clinical data, conventional models such as logistic regression, random forests, and gradient-boosting methods remain strong baselines. Intelligible models have shown competitive performance in healthcare prediction tasks (10), and XGBoost has become a widely used scalable boosting framework for tabular data modeling (11).

Recent studies have further demonstrated the feasibility of machine learning and neural-network-based approaches for chronic kidney disease (CKD) detection, healthcare outcome forecasting, and high-dimensional biomedical feature analysis (12–15). These studies support the use of data-driven models in clinical risk assessment, but they primarily focus on general CKD detection, cardiovascular risk forecasting, or feature selection methodology. They do not specifically address admission laboratory-based screening for advanced renal dysfunction in critically ill populations, nor do they incorporate clinically structured multi-branch architectures with explicit feature-interaction modeling and missingness-aware representation.

Despite these advances, several challenges remain when applying machine learning to admission laboratory data for uremia-associated advanced renal dysfunction. First, routine laboratory variables are physiologically heterogeneous: renal function markers, electrolytes, acid–base indicators, hematologic indices, and nutritional markers differ in scale, distribution, missingness pattern, and clinical interpretation. A conventional fully connected model that treats all variables as an unstructured vector may fail to preserve these domain-specific relationships. Deep learning methods can learn nonlinear representations from heterogeneous clinical variables (16–19), but their use in tabular EHR data requires clinically meaningful structure and careful validation.

Second, missing laboratory values are common in EHRs because laboratory tests are ordered according to clinical judgment rather than a fixed research protocol (20, 21). Missingness may therefore reflect both data incompleteness and clinically meaningful test-ordering behavior. Treating all missing values only as random noise may obscure informative patterns, whereas missingness indicators can preserve part of this information for downstream prediction. Therefore, a model for routine laboratory-based screening should account for both measured laboratory values and the availability pattern of those measurements.

Third, models for renal dysfunction may be vulnerable to circular reasoning if their performance is driven almost entirely by serum creatinine and BUN, which are central clinical indicators used to diagnose kidney dysfunction (22, 23). If a model achieves high discrimination primarily by leveraging these dominant renal markers, it may reproduce existing diagnostic criteria rather than extract complementary multivariable signals from the broader laboratory panel. To address this concern, such models should be evaluated against conventional machine learning baselines, clinical rule-based criteria such as eGFR thresholds, and reduced-feature models that isolate the contribution of dominant renal markers. Transparent reporting, patient-level splitting, uncertainty estimation, calibration assessment, and bias evaluation are also necessary to avoid overstating model performance (24, 25).

To address these issues, we propose MLP-CKD, a clinically informed multi-branch feature interaction-enhanced multilayer perceptron framework for admission laboratory-based screening and risk stratification of uremia-associated advanced renal dysfunction. Laboratory variables are grouped according to clinical domains, including renal function, electrolytes, acid–base status, hematologic indices, and nutritional/metabolic markers, and processed by parallel branch networks. The branch representations are then fused and passed through a feature-interaction module to model cross-domain nonlinear relationships. Missingness indicators are incorporated to preserve test-availability information, and the final model outputs an individualized risk score for admission-based screening.

Importantly, the present study is formulated as classification of admission-associated disease status using laboratory variables obtained within the first 24 h after intensive care unit (ICU) admission. It is not designed to predict future disease onset at a predefined horizon. Therefore, we avoid the terms “before onset,” “early detection,” and “future prediction” and instead use “identification,” “screening,” and “risk stratification.” This distinction is essential because the outcome label is determined at the hospital-admission level and the predictor variables are extracted from the early admission period.

The main contributions of this study are threefold. First, we constructed a reproducible MIMIC-IV-based cohort using adult ICU admissions, first-stay retention, a 24-h laboratory observation window, and patient-level training, validation, and independent test splitting to reduce information leakage from repeated admissions (6, 24). Second, we designed a clinically grouped multi-branch MLP framework with missingness-aware feature representation and a dedicated feature-interaction module to integrate heterogeneous laboratory systems while preserving domain-specific structure. Third, we evaluated the model using conventional and stronger tabular baselines, neural ablation variants, clinical rule-based baselines, bootstrap-based 95% confidence intervals, paired statistical comparisons, calibration analysis, decision curve analysis, and MLP-CKD-specific permutation importance. Through this framework, we aimed to determine whether clinically structured deep learning can improve admission laboratory-based screening of uremia-associated advanced renal dysfunction beyond standard machine learning models and simple renal-marker rules.

## 2 Materials and methods

### 2.1 Study design, data source, and cohort construction

This retrospective study was designed to develop and validate an admission laboratory-based screening model for uremia-associated advanced renal dysfunction using routinely collected electronic health record (EHR) data. The task was formulated as classification of admission-associated disease status and risk stratification, rather than prediction of future disease onset. Predictor variables were extracted from laboratory measurements obtained within the first 24 h after intensive care unit (ICU) admission, and the outcome label was defined at the hospital-admission level. This design reflects a realistic clinical scenario in which laboratory data become available shortly after ICU admission and can be used to screen for advanced renal dysfunction at the point of care.

Data were obtained from MIMIC-IV version 3.1, a publicly available de-identified EHR database distributed through PhysioNet to credentialed users who complete the required training and data-use agreement (6). This study involved secondary analysis of de-identified data and did not involve direct patient contact or intervention; therefore, additional informed consent was not required.

The cohort construction workflow proceeded in seven steps. First, all ICU stays with non-missing hospital admission identifiers were extracted. Second, stays of patients younger than 18 years were excluded. Third, only the first eligible ICU stay per patient was retained to prevent information leakage from repeated admissions. Fourth, ICU stays were linked to hospital admission records via the hospital admission identifier. Fifth, the outcome label was assigned at the hospital-admission level. Sixth, laboratory measurements were extracted from the first 24 h after ICU admission. Seventh, the cohort was split at the patient level into training, validation, and independent test sets. Key exclusion criteria included age below 18 years, repeat admissions beyond the first eligible ICU stay, missing or unlinkable hospital admission identifiers, and records with invalid laboratory extraction windows. Patients receiving renal replacement therapy or kidney transplantation were not excluded, as these procedures are part of the outcome definition and their exclusion would introduce selection bias. The full inclusion and exclusion criteria, SQL query logic, and MIMIC-IV item identifiers are provided in Supplementary Table S1.

The final analytic cohort contained 65,366 unique patients, of whom 20,074 were positive for uremia-associated advanced renal dysfunction. The cohort was split at the patient level with 70% of patients allocated to training, 15% to validation, and 15% to the independent test set, stratified by outcome label. The random seed was fixed at 42, and no patient appeared in more than one subset. The validation set was used exclusively for hyperparameter tuning, early stopping, and decision-threshold selection; the independent test set was used only for final model evaluation.

### 2.2 Outcome definition, laboratory feature construction, and preprocessing

The outcome was uremia-associated advanced renal dysfunction, defined as an operational hospital-admission-level label. A positive label was assigned if at least one of the following three criteria was met. First, the hospital admission diagnosis tables contained documented ICD-9 or ICD-10 diagnosis codes for end-stage renal disease or kidney failure. Second, the procedure records contained entries for renal replacement therapy, namely hemodialysis or peritoneal dialysis. Third, the estimated glomerular filtration rate was below 15 mL per minute per 1.73 m^2^, calculated using the CKD-EPI 2021 equation when sufficient serum creatinine and demographic data were available. The full ICD code list, procedure codes, and laboratory item identifiers are provided in Supplementary Table S2. This label should be interpreted as an operational phenotype for model development rather than as an adjudicated nephrologist-confirmed diagnosis.

Routine laboratory variables were extracted from the first 24 h after ICU admission using the laboratory event table. The selected items covered seven clinically relevant domains of advanced renal dysfunction: renal function, comprising serum creatinine and blood urea nitrogen; electrolytes, comprising sodium, potassium, and chloride; acid–base status, comprising bicarbonate and anion gap; hematologic indices, comprising hemoglobin, white blood cell count, platelet count, and hematocrit; mineral metabolism, comprising calcium, phosphate, and magnesium; glucose metabolism, comprising glucose; and nutritional status, comprising albumin. For each variable, the mean, minimum, and maximum values within the 24-h observation window were calculated to represent both the central tendency and short-term fluctuation of early admission measurements.

For each laboratory summary feature, a binary missingness indicator was generated to denote whether the corresponding measurement was observed within the 24-h window, with 1 indicating observation and 0 indicating non-observation. Missing numerical values were imputed using the median calculated exclusively from the training set, and the same imputation values were applied to the validation and test sets to prevent information leakage. Continuous variables were standardized using z-score normalization fitted on the training set and applied to the validation and test sets. Outlier management was performed during feature extraction: values exceeding the physiologically plausible ranges listed in Supplementary Table S3 were treated as missing and subsequently handled by the imputation and missingness indicator pipeline. Age and sex were also included as demographic model inputs. After completing all preprocessing steps, the final input dimension was 132 variables, comprising laboratory summary features, missingness indicators, and demographic variables. This strategy allowed all models to utilize both observed laboratory values and clinically informative test-availability patterns.

### 2.3 MLP-CKD architecture and comparator models

MLP-CKD is a clinically informed multilayer perceptron that integrates multi-branch feature grouping, a gated interaction module, and missingness-aware input representation. The central design principle is to organize laboratory variables by clinical domain before neural processing, so that physiologically related measurements are handled together rather than being treated as an undifferentiated input vector.

Clinical domain grouping and branch networks. The 132-dimensional input feature vector was partitioned into six clinical domains: renal function, electrolytes, acid–base status, hematologic indices, mineral and glucose metabolism, and nutritional status. Demographic features were assigned to a dedicated demographic branch. Let x=[x(1),,,x(2),,,…,,,x(K)] denote the partitioned input across K domains. For each domain k, a branch network $f_{k}$ learns a domain-specific latent representation $h_{k}=f_{k}(x(k))$ . Each branch consisted of two fully connected layers, with each layer followed by batch normalization, GELU activation, and dropout. The hidden dimension of each branch was selected according to the number of input features in the corresponding domain and was tuned on the validation set. The domain-specific representations were concatenated into a fused vector $h=[h_{1},,,h_{2},,,\ldots ,,,h_{K}]$ .

Feature-interaction module. The fused representation *h* was passed through a residual gated interaction module to capture nonlinear relationships across clinical domains. Two parallel projections generated a nonlinear candidate representation and a gate vector. Their element-wise product formed a gated representation, which was concatenated with the original fused vector and linearly projected. A residual connection then produced the final interaction-enhanced representation *z*. This gated residual design enables the module to selectively amplify informative cross-domain feature combinations while preserving the original branch outputs as a fallback, thereby preventing the interaction modeling from overwriting clinically meaningful domain-specific patterns.

Backbone and output. The enhanced representation *z* was fed into a backbone MLP composed of three hidden layers with 384, 192, and 96 units, respectively. Each hidden layer was followed by GELU activation, batch normalization, and dropout. The final hidden representation was mapped to a single logit through a linear output layer, and the predicted probability was obtained by sigmoid activation. This probability was interpreted as an admission-based risk score for screening and risk stratification.

Comparator models. To evaluate the contribution of each architectural design choice, MLP-CKD was compared against conventional machine learning models, neural ablation variants, and clinical rule-based comparators. Conventional baselines included logistic regression, calibrated logistic regression using Platt scaling, random forest, and XGBoost. LightGBM and CatBoost were additionally included as supplementary gradient-boosting comparators when the corresponding packages were available. Neural ablation variants included a Plain MLP without clinical grouping or feature interaction, a Branch-only MLP that retained domain-specific branches but removed the interaction module, an Interaction-only MLP that retained the gated interaction but used a single flat input, an MLP variant without missingness indicators, an MLP variant without creatinine and BUN features, and a creatinine/BUN-only model. A clinical rule-based comparator based on eGFR <15 mL/min/1.73 m^2^ was used to assess whether multivariable models provided value beyond a simple renal-function threshold. All models were evaluated using identical patient-level training, validation, and test splits.

### 2.4 Model training, evaluation, and analysis

All neural network models were trained using the AdamW optimizer. Weighted binary cross-entropy loss was employed, with the positive-class weight set inversely proportional to the training-set prevalence. Batch normalization was applied after each linear layer, and dropout was used throughout. Early stopping was based on validation-set performance, and the model weights corresponding to the best validation score were restored. Hyperparameters, including hidden-layer dimensions, dropout rates, learning rate, weight decay, and interaction-module dimensions, were selected by grid search on the validation set. For non-neural baselines, hyperparameters were selected using the same validation set via grid or randomized search. No test-set information was used during hyperparameter tuning at any stage.

Model discrimination was evaluated using the area under the receiver operating characteristic curve and the area under the precision-recall curve. Threshold-dependent metrics, including precision, recall, specificity, and F1-score, were calculated using decision thresholds selected on the validation set and then applied to the independent test set. Probability calibration was assessed using calibration curves and the Brier score. Uncertainty was estimated using bootstrap resampling of the independent test set with 1,000 iterations, and 95% confidence intervals were calculated from the 2.5th and 97.5th percentiles of the bootstrap distribution. Pairwise comparisons between MLP-CKD and each comparator were performed using paired bootstrap differences in AUC, with two-sided *p* values estimated from the empirical distribution of paired differences. A threshold of *p* < 0.05 was considered statistically significant.

Global feature importance of the trained MLP-CKD model was assessed using permutation importance on the independent test set: each feature was randomly permuted across patients, and the resulting decrease in AUC was measured. Repeated permutations were performed with 30 repetitions to estimate ranking stability, and the mean AUC decrease with standard deviation is reported for each feature. Clinical utility was evaluated using decision curve analysis across threshold probabilities ranging from 1 to 30%, with net benefit compared against treat-all and treat-none strategies. Risk stratification was assessed by grouping patients according to predicted risk scores and comparing observed outcome prevalence across strata.

All experiments were implemented in Python 3.10 using PyTorch for neural network models and scikit-learn for conventional baselines. Data extraction, preprocessing, model training, evaluation, bootstrap analysis, statistical comparison, and figure generation were performed using reproducible scripts with fixed random seeds. The MIMIC-IV data are available through PhysioNet.

## 3 Results

### 3.1 Cohort construction and patient-level splitting

The study cohort was constructed from adult ICU admissions in MIMIC-IV according to a predefined extraction workflow. Patients aged 18 years or older were eligible for inclusion. To avoid information leakage from repeated admissions, only the first eligible ICU stay was retained for each patient before model development and evaluation. The outcome label was defined at the hospital-admission level according to the predefined uremia-associated advanced renal dysfunction criteria, and routine laboratory variables were extracted from the first 24 h after ICU admission.

Because the laboratory window was anchored to ICU admission and the outcome label was determined at the admission level, the task was formulated as admission laboratory-based identification and risk stratification of uremia-associated advanced renal dysfunction, rather than prediction of future disease onset. The final cohort was split at the patient level into training, validation, and independent test sets. The split was stratified by outcome label, and the random seed was fixed to ensure reproducibility. No patient appeared in more than one subset.

**Figure 1:**
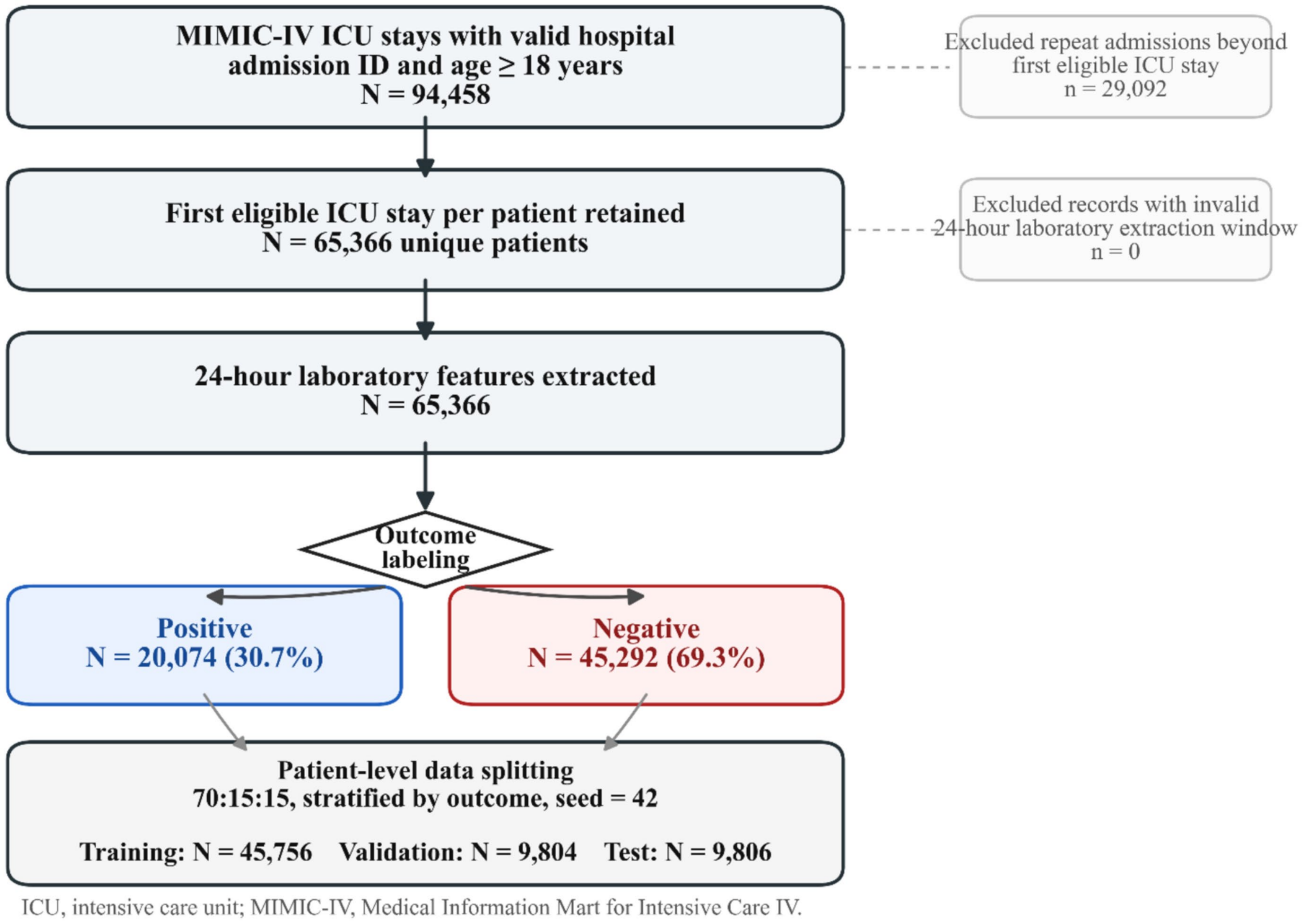
Cohort construction workflow and patient-level data splitting. Adult ICU stays were screened, repeated admissions were handled by retaining the first eligible ICU admission per patient, laboratory variables were extracted within the first 24 h after ICU admission, and the final cohort was split at the patient level into training, validation, and independent test sets.

The cohort selection process is summarized in Table 1 and illustrated in the cohort flow diagram. This workflow documents the transition from the source MIMIC-IV population to the final analytic cohort, including adult selection, first-stay retention, laboratory window availability, and patient-level train-validation-test splitting. This design reduces the risk of record-level leakage and provides a reproducible basis for subsequent model comparison (see Figure 1).

**Table 1:** Cohort construction and patient-level sample flow.

Cohort construction step	Number
ICU stays with non-missing hospital admission ID	94,458
Adult ICU stays with age > = 18	94,458
Admission handling: first_patient	65,366
Unique patients in final cohort before laboratory filtering	65,366
Records with extracted 24-h laboratory feature table	65,366
Positive records by operational outcome label	20,074
Negative records by ICD-based label	45,292

### 3.2 Baseline characteristics and laboratory missingness

Baseline demographic and laboratory characteristics are summarized in Table 2. Continuous variables are reported as medians with interquartile ranges, and categorical variables are reported as counts and percentages. Compared with the non-case group, patients with uremia-associated advanced renal dysfunction showed higher levels of renal function-related markers, especially serum creatinine and blood urea nitrogen, together with abnormalities in electrolyte, acid–base, hematologic, and nutritional indicators. These patterns are consistent with the expected clinical profile of advanced renal impairment.

**Table 2:** Baseline characteristics of cases and non-cases.

Variable	Case	Non-case	Missing (%)
Age	68.00 (56.00–79.00)	64.00 (52.00–75.00)	0.0
albumin_mean	3.00 (2.50–3.40)	3.30 (2.80–3.80)	69.2
bicarbonate_mean	22.20 (19.33–25.00)	24.00 (22.00–26.00)	2.4
bun_mean	35.00 (22.00–61.33)	15.00 (11.00–20.00)	2.7
creatinine_mean	1.80 (1.20–5.60)	0.80 (0.67–1.00)	2.7
Gender	11,998 (59.8%)	24,722 (54.6%)	0.0
hemoglobin_mean	9.80 (8.50–11.35)	10.78 (9.40–12.20)	3.1
phosphate_mean	3.90 (3.10–4.92)	3.30 (2.80–3.80)	8.9
potassium_mean	4.35 (3.90–4.95)	4.10 (3.80–4.40)	2.5
sodium_mean	137.00 (132.67–140.00)	138.20 (136.00–140.50)	2.5

The laboratory variables used for model development are listed in Table 3. For each laboratory item, summary statistics within the first 24 h were extracted, including mean, minimum, and maximum values. Binary missingness indicators were additionally generated to preserve information carried by test-ordering patterns. The final feature set therefore contained demographic variables, laboratory summary features, and missingness flags. This design allowed the models to use both observed laboratory values and missing-data patterns, which are common in real-world electronic health record data.

**Table 3:** Laboratory variables, clinical categories, missingness rates, and input-use status.

Variable	Base variable	Clinical category	Missing *n*	Missing (%)	Used as input
Age	Age	Demographic	0	0.0	Yes
Gender	Gender	Demographic	0	0.0	Yes
creatinine_mean	Creatinine	renal_function	2,040	3.1	Yes
creatinine_min	Creatinine	renal_function	2,040	3.1	Yes
creatinine_max	Creatinine	renal_function	2,040	3.1	Yes
creatinine_count	Creatinine	renal_function	2,040	3.1	Yes
bun_mean	Bun	renal_function	2,076	3.2	Yes
bun_min	Bun	renal_function	2,076	3.2	Yes
bun_max	Bun	renal_function	2,076	3.2	Yes
bun_count	Bun	renal_function	2,076	3.2	Yes
sodium_mean	Sodium	electrolyte_acid_base	1,956	3.0	Yes
sodium_min	Sodium	electrolyte_acid_base	1,956	3.0	Yes
sodium_max	Sodium	electrolyte_acid_base	1,956	3.0	Yes
sodium_count	Sodium	electrolyte_acid_base	1,956	3.0	Yes
potassium_mean	Potassium	electrolyte_acid_base	1,937	3.0	Yes
potassium_min	Potassium	electrolyte_acid_base	1,937	3.0	Yes
potassium_max	Potassium	electrolyte_acid_base	1,937	3.0	Yes
potassium_count	Potassium	electrolyte_acid_base	1,937	3.0	Yes
chloride_mean	Chloride	electrolyte_acid_base	1,986	3.0	Yes
chloride_min	Chloride	electrolyte_acid_base	1,986	3.0	Yes

The missingness analysis further showed that laboratory availability varied across clinical domains. Renal function markers were more consistently observed, whereas some metabolic, hematologic, and nutritional variables had higher missingness. These findings support the inclusion of missingness indicators rather than treating missing data only as a nuisance to be imputed.

### 3.3 Model architecture and experimental settings

The proposed MLP-CKD architecture is summarized in Table 4 and illustrated in the model architecture diagram. Laboratory variables were first grouped according to clinical domains, including renal function, electrolytes, acid–base balance, hematologic markers, and metabolic or nutritional indicators. Each group was processed by a dedicated branch network to learn domain-specific representations. The branch outputs were then concatenated and passed through a fusion layer and feature-interaction module, followed by a backbone multilayer perceptron and a sigmoid output layer.

**Table 4:** Architecture of the proposed MLP-CKD model.

Component	Details	Activation	Dropout
Input	132 variables after imputation, standardization, and optional missingness indicators	—	—
Clinical branches	6 branches; each branch: Linear → BN → GELU → Dropout → Linear → BN → GELU → Dropout	GELU	0.05–0.15 tuned
Fusion layer	Concatenation of branch representations followed by dense projection	GELU	0.05–0.15 tuned
Interaction module	Residual gated interaction: tanh(Wa h) * sigmoid(Wb h), concatenated with h, projected, then added back to h	tanh/sigmoid/GELU	0.05–0.15 tuned
Backbone	Tuned fully connected layers, default candidate: 384 → 192 → 96	GELU + BN	0.05–0.15 tuned
Output	Single logit with sigmoid probability at inference	Sigmoid	—

**Figure 2:**
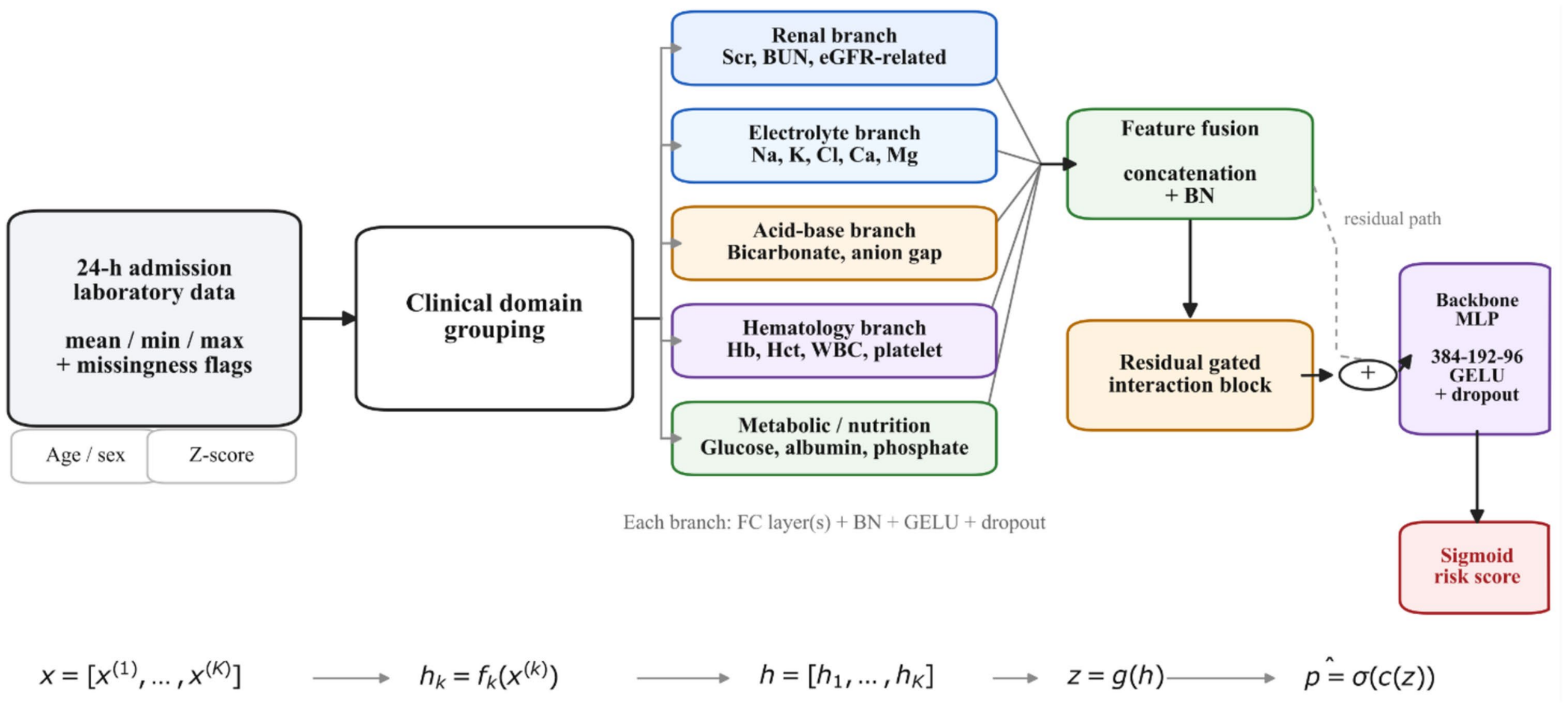
Architecture of the proposed MLP-CKD framework. Clinically related laboratory variables were assigned to parallel branches, fused into a shared representation, processed through a residual gated interaction module, and passed to a fully connected backbone for probability estimation.

All neural network models were trained on the training set, and hyperparameters were selected using the validation set. The independent patient-level test set was used only for final evaluation. Model performance was assessed using discrimination metrics, threshold-dependent classification metrics, and calibration metrics. AUC and AUPRC were used as primary discrimination metrics. Precision, recall, specificity, and F1-score were calculated at validation-selected thresholds. Brier score was used to assess probability calibration. Uncertainty was estimated using bootstrap resampling of the independent test set (see Figure 2).

### 3.4 Main model comparison on the independent patient-level test set

The primary quantitative model comparison is shown in Table 5. The main comparison included logistic regression, calibrated logistic regression, random forest, XGBoost, Plain MLP, Branch-only MLP, Interaction-only MLP, and the proposed MLP-CKD. For each model, AUC, AUPRC, precision, recall, specificity, F1-score, Brier score, and 95% confidence intervals were reported.

**Table 5:** Main model performance on the independent patient-level test set.

Model	AUC (95% CI)	AUPRC (95% CI)	Precision (95% CI)	Recall (95% CI)	Specificity (95% CI)	F1 (95% CI)	Brier (95% CI)	Threshold
MLP-CKD	0.913 (0.907-0.919)	0.848 (0.838–0.859)	0.734 (0.719–0.749)	0.796 (0.783–0.811)	0.872 (0.864–0.880)	0.764 (0.752–0.775)	0.121 (0.117–0.125)	0.59
Branch–only MLP	0.913 (0.907–0.919)	0.846 (0.835–0.857)	0.714 (0.699–0.729)	0.819 (0.806–0.833)	0.855 (0.846–0.863)	0.763 (0.751–0.775)	0.113 (0.109–0.116)	0.49
Interaction-only MLP	0.911 (0.905–0.917)	0.845 (0.833–0.856)	0.728 (0.713–0.744)	0.804 (0.791–0.818)	0.867 (0.858–0.875)	0.764 (0.754–0.777)	0.118 (0.114–0.122)	0.54
Plain MLP	0.910 (0.904–0.916)	0.844 (0.833–0.855)	0.733 (0.718–0.749)	0.793 (0.781–0.808)	0.872 (0.864–0.880)	0.762 (0.751–0.774)	0.116 (0.112–0.119)	0.55
XGBoost	0.903 (0.897–0.909)	0.824 (0.810–0.837)	0.752 (0.736–0.768)	0.753 (0.738–0.768)	0.890 (0.883–0.897)	0.752 (0.740–0.765)	0.124 (0.120–0.128)	0.63
Random Forest	0.897 (0.890–0.903)	0.815 (0.801–0.828)	0.701 (0.685–0.717)	0.799 (0.786–0.813)	0.849 (0.841–0.858)	0.747 (0.735–0.759)	0.134 (0.131–0.137)	0.52
Calibrated LR	0.902 (0.895–0.908)	0.825 (0.814–0.838)	0.739 (0.724–0.754)	0.763 (0.748–0.778)	0.881 (0.873–0.888)	0.751 (0.739–0.762)	0.109 (0.106–0.113)	0.39
LR	0.901 (0.895–0.908)	0.827 (0.815–0.839)	0.743 (0.728–0.758)	0.758 (0.744–0.774)	0.884 (0.876–0.891)	0.750 (0.739–0.762)	0.121 (0.117–0.125)	0.52

Among the primary models in Table 5, MLP-CKD achieved the highest AUC and AUPRC. This indicates that the clinically informed multi-branch feature-interaction architecture provided the strongest overall discrimination for admission laboratory-based identification of uremia-associated advanced renal dysfunction. Compared with conventional machine learning baselines, MLP-CKD preserved competitive discrimination while maintaining a screening-oriented sensitivity profile, suggesting potential utility for identifying patients who may require further nephrology evaluation.

**Figure 3:**
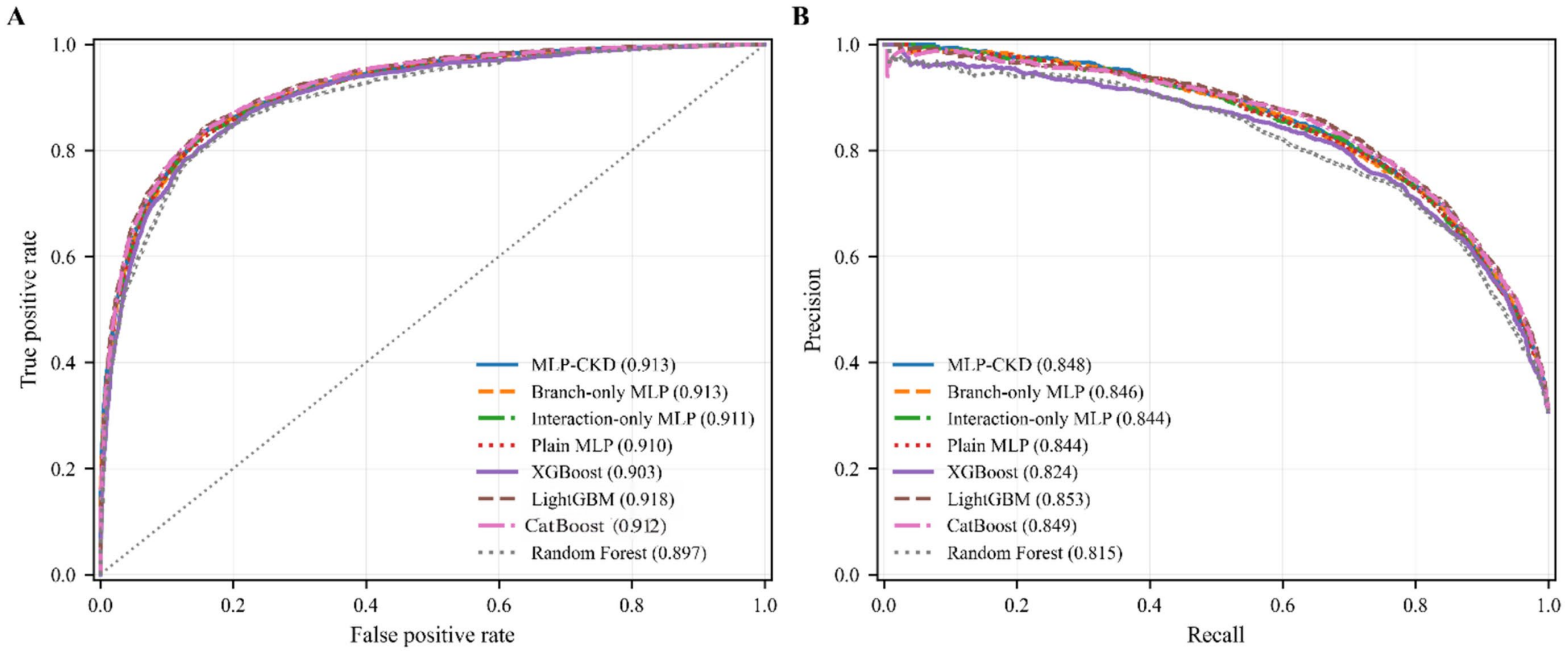
Receiver operating characteristic and precision–recall curves on the independent patient‑level test set. **(A)** ROC curves generated from patient‑level predicted probabilities. In addition to the primary models in Table 5, LightGBM and CatBoost were included as supplementary curve‑level comparators. **(B)** Precision–recall curves for the same set of models. The main quantitative statistics with 95% confidence intervals are reported in Table 5.

Figure 3 shows the ROC and precision–recall curves generated from patient-level predicted probabilities on the independent test set. In addition to the primary models summarized in Table 5, LightGBM and CatBoost were included in Figure 3 as supplementary strong gradient-boosting baselines for visual comparison. The curves show that MLP-CKD remained competitive against both conventional classifiers and modern boosting-based tabular models. Because LightGBM and CatBoost were included as curve-level comparators but were not part of the current main 95% confidence interval summary table, the principal quantitative claims are based on Table 5 and the paired bootstrap analysis in Table 6.

**Table 6:** Pairwise paired-bootstrap AUC comparisons between MLP-CKD and comparator models.

Reference model	Compared model	Delta AUC	Paired bootstrap P
MLP-CKD	LR	0.0114	<0.001
MLP-CKD	Calibrated LR	0.0113	<0.001
MLP-CKD	Random forest	0.0161	<0.001
MLP-CKD	XGBoost	0.0098	<0.001
MLP-CKD	Plain MLP	0.0026	0.022
MLP-CKD	Branch-only MLP	0.0001	0.952
MLP-CKD	Interaction-only MLP	0.0022	0.050
MLP-CKD	Creatinine/BUN-only LR	0.0183	<0.001
MLP-CKD	Without Scr/BUN LR	0.0893	<0.001
MLP-CKD	Without missingness LR	0.011	<0.001
MLP-CKD	eGFR rule	0.0474	<0.001

The improvement of MLP-CKD over the Branch-only MLP was modest. This finding suggests that clinically informed feature grouping captured a substantial proportion of the discriminative signal, while the interaction module provided additional but limited incremental benefit. Therefore, the proposed model should be interpreted as a structured enhancement of a clinically grouped MLP rather than as a model that overwhelmingly outperforms all ablation variants.

### 3.5 Ablation study and clinical rule-based baseline comparison

**Figure 4:**
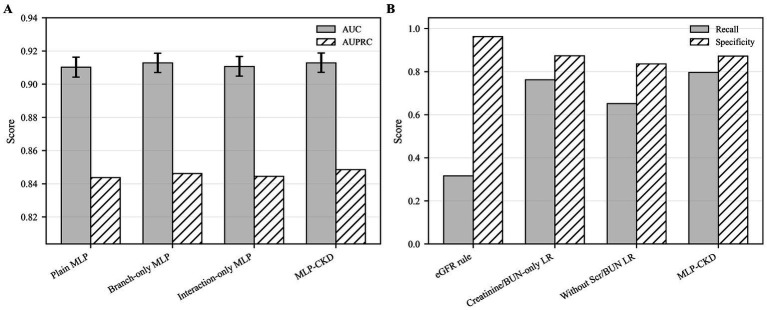
Ablation analysis and clinical rule‑based baseline comparison. **(A)** Area under the receiver operating characteristic curve (AUC) with 95% bootstrap confidence intervals for MLP‑CKD and the ablation/rule‑based comparators, including plain MLP, branch‑only MLP, interaction‑only MLP, feature‑restricted baselines, and the eGFR rule. **(B)** Area under the precision–recall curve (AUPRC) for the same models. Numerical values are provided in Table 6.

Ablation results are summarized in Table 7 and Figure 4. Plain MLP served as the basic fully connected neural network baseline. Branch-only MLP retained the clinical feature grouping strategy but removed the interaction module. Interaction-only MLP retained the interaction component without the full clinically grouped branch structure. The complete MLP-CKD model combined clinical grouping, feature fusion, and interaction modeling.

**Table 7:** Ablation analysis and clinical rule-based baseline comparison.

Model	AUC (95% CI)	AUPRC (95% CI)	Recall (95% CI)	Specificity (95% CI)	F1 (95% CI)	Brier (95% CI)
MLP-CKD	0.913 (0.907–0.919)	0.848 (0.838–0.859)	0.796 (0.783–0.811)	0.872 (0.864–0.880)	0.764 (0.752–0.775)	0.121 (0.117–0.125)
Branch-only MLP	0.913 (0.907–0.919)	0.846 (0.835–0.857)	0.819 (0.806–0.833)	0.855 (0.846–0.863)	0.763 (0.751–0.775)	0.113 (0.109–0.116)
Interaction-only MLP	0.911 (0.905–0.917)	0.845 (0.833–0.856)	0.804 (0.791–0.818)	0.867 (0.858–0.875)	0.764 (0.754–0.777)	0.118 (0.114–0.122)
Plain MLP	0.910 (0.904–0.916)	0.844 (0.833–0.855)	0.793 (0.781–0.808)	0.872 (0.864–0.880)	0.762 (0.751–0.774)	0.116 (0.112–0.119)
Without missingness LR	0.902 (0.896–0.909)	0.827 (0.815–0.839)	0.768 (0.753–0.784)	0.875 (0.868–0.883)	0.749 (0.738–0.762)	0.121 (0.118–0.125)
Creatinine/BUN-only LR	0.895 (0.888–0.902)	0.820 (0.808–0.832)	0.762 (0.748–0.778)	0.874 (0.865–0.881)	0.744 (0.732–0.756)	0.125 (0.121–0.128)
eGFR rule	0.866 (0.858–0.874)	0.707 (0.686–0.726)	0.316 (0.300–0.334)	0.963 (0.958–0.967)	0.452 (0.432–0.471)	0.218 (0.210–0.225)
Without Scr/BUN LR	0.824 (0.815–0.833)	0.708 (0.692–0.725)	0.652 (0.636–0.669)	0.836 (0.826–0.844)	0.644 (0.631–0.659)	0.166 (0.162–0.169)

The Branch-only MLP achieved performance close to that of MLP-CKD, indicating that clinically informed grouping of laboratory variables was the main contributor to the neural model’s performance. The full MLP-CKD model showed the best or near-best discrimination, but the incremental gain over the branch-only variant was small. This pattern suggests that the interaction module may refine the fused representation, but its contribution should be described as incremental rather than dominant.

Additional ablation experiments examined whether the model merely reproduced known renal function criteria. The creatinine/BUN-only model achieved strong performance but remained inferior to the complete MLP-CKD model. Conversely, the model without creatinine and BUN retained moderate discriminative ability. These results indicate that creatinine and BUN were dominant predictors, but the model did not rely exclusively on these two markers. Instead, electrolyte, acid–base, hematologic, and nutritional variables provided complementary information.

The eGFR rule-based baseline showed high specificity but limited recall. This pattern is consistent with the behavior of a strict clinical rule: it identifies a subset of clear advanced renal dysfunction cases but misses patients with more complex or incomplete laboratory profiles. In contrast, multivariable machine learning models improved sensitivity by integrating broader laboratory patterns. These findings support the use of MLP-CKD as a screening and risk stratification tool rather than as a replacement for clinical diagnosis.

### 3.6 Statistical uncertainty and pairwise model comparison

To quantify uncertainty, 95% confidence intervals were estimated using bootstrap resampling of the independent patient-level test set. Pairwise model comparisons were further performed using paired bootstrap differences in AUC, with MLP-CKD as the reference model. Results are summarized in Table 6.

MLP-CKD showed improved discrimination compared with several conventional baseline models. However, the difference between MLP-CKD and the Branch-only MLP was small and not statistically significant. This result is important for interpretation: it indicates that the proposed full model reached the highest overall discrimination, but the evidence for superiority over its strongest ablation variant was limited. Therefore, the experimental conclusion should emphasize competitive performance and the value of clinically informed representation learning rather than claiming broad statistical superiority over all neural variants.

This uncertainty analysis also provides a more balanced interpretation of model ranking. Small differences in AUC or AUPRC should not be over-interpreted when confidence intervals overlap or paired bootstrap tests are non-significant. Accordingly, MLP-CKD is positioned as a high-performing and clinically structured screening model, with its main advantage lying in combining competitive discrimination, sensitivity-oriented behavior, and interpretable laboratory-domain organization.

### 3.7 Calibration and clinical utility analysis

**Figure 5:**
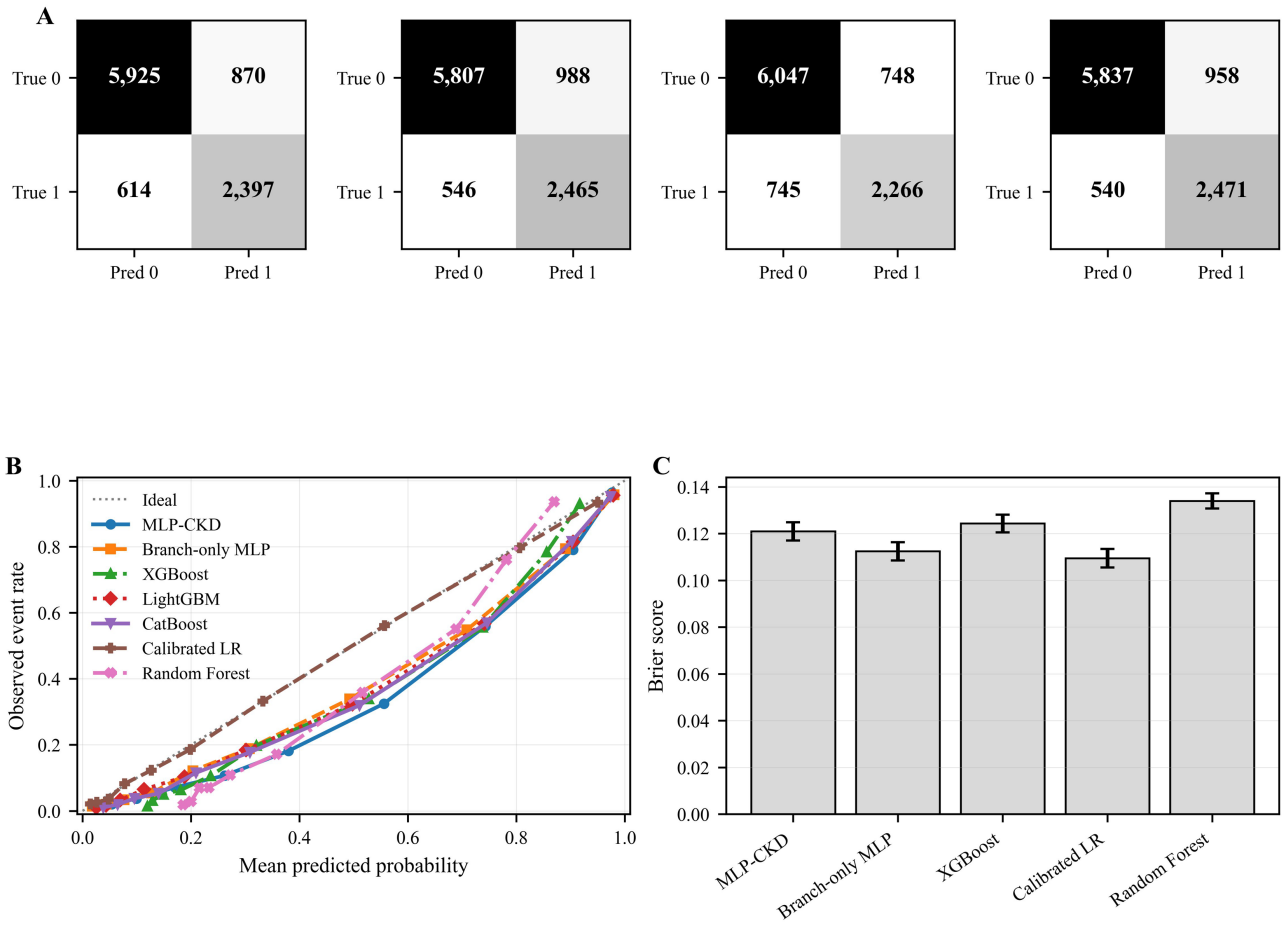
Confusion matrices, calibration curves, and Brier‑score comparison. Thresholds were selected on the validation set and applied to the independent test set. **(A)** Confusion matrices for the evaluated models showing true positive, false positive, true negative, and false negative counts. **(B)** Calibration curves comparing predicted probabilities against observed outcome frequencies. LightGBM and CatBoost are shown as supplementary visual comparators. **(C)** Brier‑score comparison, with lower values indicating better probability calibration.

Figure 5 compares threshold-dependent classification behavior and probability calibration. The confusion matrices show that the models differed in their balance between missed positive cases and false-positive alerts. MLP-CKD demonstrated a screening-oriented operating pattern, consistent with the clinical aim of identifying patients who may require further renal assessment based on admission laboratory results.

Calibration analysis provided complementary information beyond discrimination. Although MLP-CKD achieved strong discriminative performance, its calibration was not uniformly superior to calibrated logistic regression or some tree-based baselines. This suggests that *post-hoc* probability calibration may be required before the predicted probabilities are used as absolute risk estimates in clinical deployment. LightGBM and CatBoost were also shown in Figure 5 as supplementary visual comparators for calibration-level assessment, whereas the main numerical calibration statistics were reported in the performance table.

**Figure 6:**
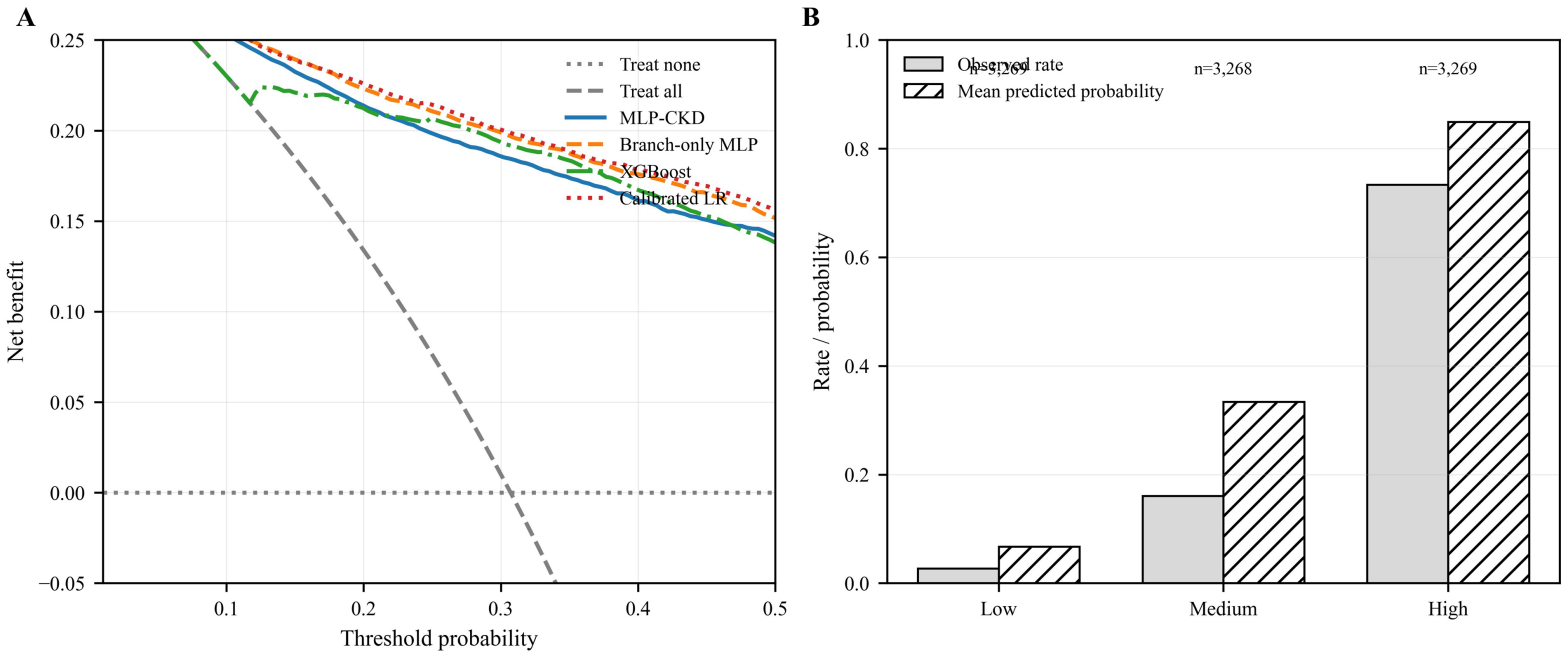
Decision curve analysis and risk stratification. **(A)** Decision curve analysis displaying net benefit across a clinically relevant range of threshold probabilities for MLP‑CKD and comparator strategies. **(B)** Risk stratification plot showing observed case prevalence (proportion of uremia‑associated advanced renal dysfunction) across predicted‑risk strata.

Clinical utility was further evaluated using decision curve analysis and risk stratification, as shown in Figure 6. The decision curve indicated that MLP-CKD provided positive net benefit across a clinically relevant range of threshold probabilities compared with treat-all and treat-none strategies. Risk stratification analysis showed that the observed prevalence increased across predicted risk groups, indicating that the model output could separate patients into clinically meaningful risk strata. These findings support the potential use of MLP-CKD as an admission-based screening aid, particularly for prioritizing patients who may benefit from closer renal evaluation.

### 3.8 Global feature importance of MLP-CKD

**Figure 7:**
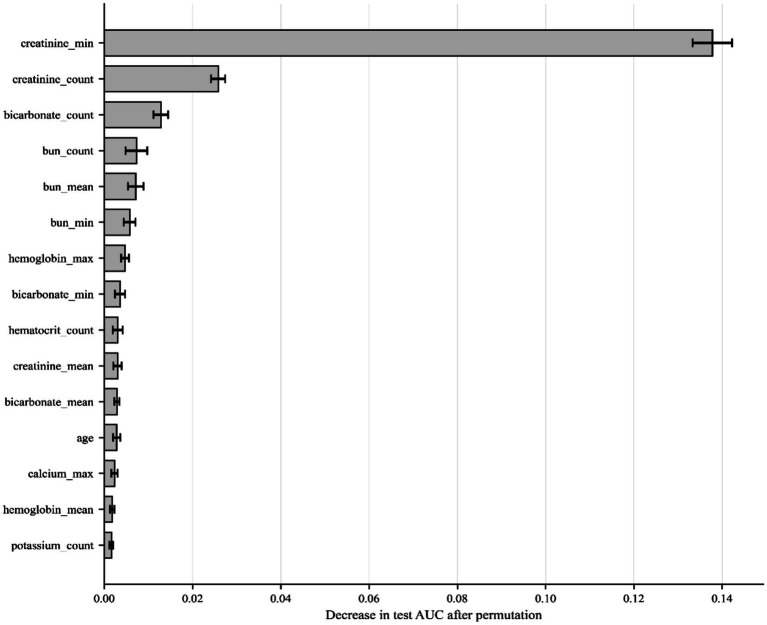
Global feature importance of MLP-CKD based on permutation importance. Each feature was permuted on the independent test set, and importance was quantified as the decrease in test AUC. Bars indicate mean AUC decrease across repeated permutations, with larger values indicating greater contribution to model performance.

Global feature importance of MLP-CKD was assessed using permutation importance on the independent patient-level test set. For each feature, values were randomly permuted and the resulting decrease in test AUC was measured. Larger decreases indicated stronger contribution to the predictive performance of MLP-CKD. The top 15 features are shown in Figure 7.

Creatinine-related features ranked among the most important predictors, confirming that renal filtration markers were central to the model’s decisions. Blood urea nitrogen-related features also contributed strongly, consistent with the role of nitrogenous waste retention in advanced renal dysfunction. Importantly, several non-renal-function features, including acid–base, hematologic, electrolyte, and demographic variables, also appeared among the top-ranked predictors. This pattern supports the ablation findings: although creatinine and BUN were dominant, MLP-CKD integrated complementary laboratory information rather than simply reproducing a single-marker diagnostic rule.

The use of permutation importance directly evaluates the trained MLP-CKD model rather than a surrogate model. Therefore, Figure 7 provides model-specific evidence for how the proposed network used laboratory variables in the admission-based screening task. The results are clinically plausible and align with known manifestations of advanced renal dysfunction, including impaired filtration, uremic solute accumulation, acid–base disturbance, anemia, and systemic metabolic imbalance.

## 4 Discussion

### 4.1 Principal findings

This study developed and evaluated MLP-CKD, a clinically informed multi-branch deep learning framework for admission laboratory-based screening and risk stratification of uremia-associated advanced renal dysfunction. The revised analysis addressed several methodological issues that are critical for clinical prediction modeling, including operational outcome definition, first-stay retention, patient-level data splitting, missingness-aware feature construction, ablation testing, clinical rule-based comparison, bootstrap uncertainty estimation, calibration assessment, decision curve analysis, and model-specific permutation importance.

On the independent patient-level test set, MLP-CKD achieved the highest discrimination among the primary models, with an AUC of 0.913 and an AUPRC of 0.848. The model outperformed conventional machine learning baselines such as logistic regression, random forest, and XGBoost in paired-bootstrap AUC comparisons. However, the difference between MLP-CKD and the Branch-only MLP was negligible and not statistically significant. This finding is important because it indicates that the principal performance gain was driven by clinically informed feature grouping, whereas the residual gated interaction module provided an incremental rather than dominant contribution.

### 4.2 Interpretation of the architecture and ablation findings

The ablation experiments provide a more cautious and mechanistic interpretation of the proposed architecture. The strong performance of the Branch-only MLP suggests that organizing heterogeneous laboratory variables into clinically meaningful domains preserves useful physiological structure. This is consistent with the clinical reality that renal function markers, electrolytes, acid–base indicators, hematologic measurements, and nutritional variables reflect different but related aspects of advanced kidney dysfunction. By contrast, the modest improvement from the interaction module suggests that cross-domain interaction modeling may refine the learned representation but should not be presented as the sole source of model improvement.

The reduced-feature analyses also help address the concern of circular reasoning. Creatinine- and BUN-related variables were dominant predictors, as expected for an outcome related to advanced renal dysfunction. Nevertheless, the creatinine/BUN-only model performed worse than the complete MLP-CKD model, and the model without creatinine and BUN retained moderate discrimination. These results suggest that the model did not merely reproduce single-marker diagnostic rules. Instead, it integrated complementary information from acid–base status, hematologic indices, electrolyte balance, nutritional markers, and missingness patterns.

### 4.3 Clinical interpretation and potential utility

The current model should be interpreted as an admission-based screening and risk stratification tool rather than as a diagnostic replacement. Its inputs were extracted from the first 24 h after ICU admission, and the outcome was defined at the hospital-admission level. Therefore, the study does not establish a future prediction horizon or demonstrate prediction before disease onset. Within this scope, the model may help prioritize patients who warrant closer renal assessment, particularly in settings where multiple laboratory abnormalities need to be interpreted simultaneously.

Calibration and clinical utility analyses provided information beyond discrimination. MLP-CKD achieved strong ranking performance, but its Brier score was not uniformly superior to calibrated logistic regression or some tree-based models. This indicates that post-hoc calibration may be required before predicted probabilities are used as absolute risk estimates. Decision curve analysis and risk stratification suggested potential net benefit and clinically meaningful separation of risk strata, but these findings should be validated prospectively before deployment in clinical workflows.

### 4.4 Model interpretation

Permutation importance directly assessed the trained MLP-CKD model rather than a surrogate model. Creatinine-related features and BUN-related features contributed most strongly to model performance, which is clinically plausible given their central role in impaired renal filtration and nitrogenous waste retention. The presence of additional acid–base, hematologic, electrolyte, and demographic variables among the important features supports the interpretation that MLP-CKD used broader laboratory patterns. These associations should be interpreted as predictive rather than causal, because the retrospective design cannot establish mechanistic causation.

### 4.5 Limitations and future work

Several limitations should be acknowledged. First, this was a retrospective study based on a single public database. Although MIMIC-IV provides detailed ICU data and enables reproducible analysis, its patient population, testing practices, and treatment workflows may not generalize to other hospitals or healthcare systems. External validation in independent and multi-center cohorts is required before clinical adoption. Second, the outcome was an operational phenotype based on coded diagnoses, renal replacement therapy-related records, and laboratory-derived criteria when available. It was not adjudicated by nephrologists and should not be considered a definitive clinical gold standard.

Third, the model used laboratory summaries from the first 24 h after ICU admission and did not incorporate longitudinal trajectories, medication data, vital signs, urine output, imaging findings, or clinician notes. These data sources may improve characterization of kidney disease status and future risk. Fourth, although patient-level splitting and first-stay retention reduced leakage, residual confounding and coding bias remain possible in retrospective EHR studies. Fifth, the improvement over the Branch-only MLP was small, which limits the strength of claims regarding the interaction module. Future work should evaluate whether the interaction module provides larger benefits in external cohorts, smaller feature sets, or longitudinal prediction tasks.

Future research should focus on external validation, prospective evaluation, and integration with broader clinical information. A clinically deployed version of MLP-CKD would also require calibration monitoring, subgroup fairness analysis, workflow integration, and assessment of whether model-guided screening improves nephrology referral, diagnostic timeliness, or patient outcomes.

## 5 Conclusion

This study presented MLP-CKD, a clinically informed multi-branch deep learning framework for admission laboratory-based screening and risk stratification of uremia-associated advanced renal dysfunction using routinely collected laboratory data. On the independent patient-level test set, MLP-CKD achieved the highest discrimination among the primary models and showed clinically plausible feature-importance patterns.

The revised ablation analyses indicate that clinically informed feature grouping was the main contributor to performance, whereas the residual gated interaction module provided additional but modest benefit. Feature-restricted and rule-based comparisons showed that the model incorporated information beyond creatinine and BUN alone, although these renal markers remained dominant predictors.

Because this retrospective study used admission-window laboratory variables and did not define a future prediction horizon, MLP-CKD should not be interpreted as a model for predicting disease onset or as a standalone diagnostic system. Rather, it provides a decision-support risk score that may assist admission-based screening and risk stratification. External validation, prospective evaluation, and calibration monitoring are needed before clinical deployment.

## Data Availability

Publicly available datasets were analyzed in this study. This data can be found at: https://physionet.org/content/mimiciv/3.1/.
