# Supplementary material for: MLP-CKD: a clinically informed deep learning framework for admission laboratory-based screening and risk stratification of uremia-associated advanced renal dysfunction
Source: Front Public Health. 2026 Jun 9;14:1858083. doi: 10.3389/fpubh.2026.1858083 (PMC13287141; doi:10.3389/fpubh.2026.1858083)
Supplement: Supplementary file 1 [file Supplementary_file_1.pdf]

Authorship change

For changes to the authorship of manuscripts, Frontiers requires that all authors complete and sign the Authorship Change form below. This includes any author(s) being added or removed, as well as all other authors included on the original author list. Signing this form indicates consent to the change(s), and agreement with the new Author Contributions, Conflict of Interest and Acknowledgment sections. Ensure that every section of this form is completed.

Authorship criteria

In accordance with the International Committee of Medical Journal Editors (ICMJE) guidelines, in order to be included in the author list a contributor must meet all 4 of the following criteria:

- Substantial contributions to the conception or design of the work; or the acquisition, analysis, or interpretation of data for the work; AND
- Drafting the work or revising it critically for important intellectual content; AND
- Final approval of the version to be published; AND
- Agreement to be accountable for all aspects of the work in ensuring that questions related to the accuracy or integrity of any part of the work are appropriately investigated and resolved.

Any contributors who meet fewer than all 4 of the above criteria for authorship must not be listed as authors, but their contributions should be detailed in the Acknowledgments section. Requests to add authors who do not meet all 4 of the above criteria will not be considered. We ask authors to use the CRediT taxonomy to clearly describe their contributions to papers. Please see CRediT for more details.

If you have any questions regarding authorship, contact the Research Integrity team of the relevant journal.

Please note that we cannot proceed with the publication of your manuscript until any outstanding authorship issues have been resolved.

Completing and signing the form

- Complete and submit an Authorship Change form if you: add, remove, or change the order of the authors after your article is accepted.
- We only accept forms written in English, signatures can be provided in Arabic and Chinese.
- Complete the form with all article and author information, and new statements (on Page 3), if they have changed.
- Complete the last page of the form with the final author list that will be used in the published article.
- The form must be signed individually, by each author.
- The form should be manually signed or contain valid e-signatures (an electronic version of a signature that can be uniquely identified and linked to the signatory, as shown in the example below).
- Useful link: [helpx.adobe.com/acrobat/using/signing-pdfs.html.](helpx.adobe.com/acrobat/using/signing-pdfs.html)
- Authors can submit signatures across different files individually if the main information is completed and all signatures are provided.

Acceptable signatures

- Digital stylus.
- Adobe “drawn” (using the default fonts) and “signed” signatures.
- Signature stamps or seals.
- Handwritten signatures.

Electronic signature

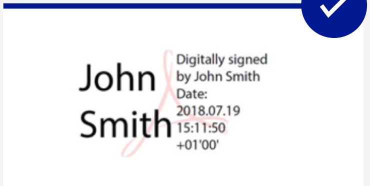

Digital stylus signature

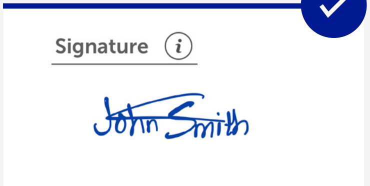

Scanned signature

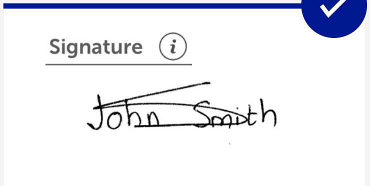

Unacceptable signatures

- Typed signatures which are not e-signatures will not be accepted.
- Signatures which have been signed on behalf of other authors/not individually signed.
- Photographs of signatures will also not be allowed.

If you provide the form with invalid signatures, we will not be able to accept it or be able to consider your request for an authorship change.

Typed signature

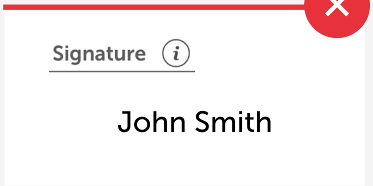

Photograph/screenshot signature

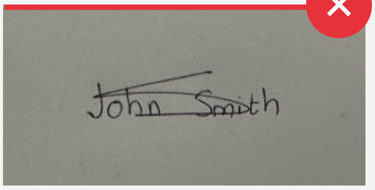

Authorship change

对于稿件作者身份的变更，Frontiers要求所有作者填写并签署下方的"作者身份变更表".任何被添加或移除的作者，以及在原始作者列表中所包含的其他所有作者均需进行填写并签署. 签署此表格即表示同意进行更改，并认可新的作者贡献、利益冲突及致谢部分. 请确保填妥此表格的每个部分.

作者身份标准

根据国际医学期刊编辑委员会（ICMJE）指导方针，文章贡献者须同时符合以下4项标准才能被列入作者名单：

- S对该项工作的构思或设计做出重大贡献；或为工作获取、分析或解析数据；
- 起草文稿或对重要的学术内容进行重大修改；
- 最终批准待发表的版本；
- 同意对该项工作的所有方面负责，以确保与工作的任何部分相关的准确性或完整性问题都能得到适当地调查和解决.

任何不符合上述全部4项作者身份标准的贡献者不得被列为作者，但贡献者的贡献应在致谢部分进行详细说明。不符合上述全部4项标准的作者添加申请将不予考虑.

如果您对作者身份有任何疑问，请随时联系相关期刊的研究诚信团队.

请注意，在任何有关作者身份的问题未得到解决前，我们无法发表您的稿件.

填写并签署表格

- 如果您在文章接收后，需要：添加、删除或更改作者的顺序，请填写并提交作者身份变更表.
- 请您用英文填写表格，签名可以是中文.
- 请将所有文章和作者的信息填写在表格中；如果添加或者删除作者，请在第3页填写新的声明.
- 在表格的最后一页填写最终作者名单，该名单将用于在线发表版本中.
- 表格必须由每位作者单独签名.
- 表格必须提供手写签名或包含有效的电子签名（可以唯一识别并链接到签名人的电子版签名，如下例所示）.
- 有效的链接：[helpx.adobe.com/acrobat/using/signing-pdfs.html.](https://helpx.adobe.com/acrobat/using/signing-pdfs.html)
- 如果作者因地域原因不能同时在同一个文件签名，可分别在不同文件填写完整信息，并单独完成签名.

可接受的签名

- 数字化签名.
- Adobe（使用默认字体）“绘制（drawn）”和“签署（signed）”的签名.
- 印章签名.
- 手写签名.

Electronic signature

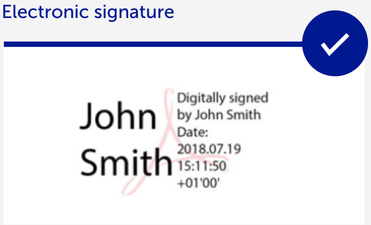

Digital stylus signature

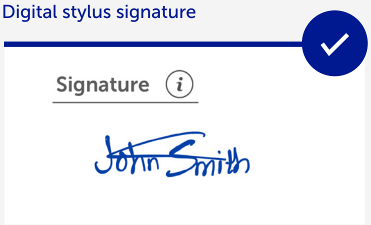

Scanned signature

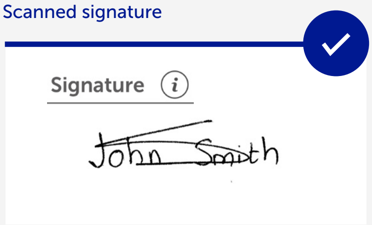

不可接受的签名

- 不接受非电子签名的打字签名.
- 代表其他作者签署的签名/非独立完成的签名.

如果您提供的表格签名无效，我们将无法接受或考虑您更改作者身份的请求

Typed signature

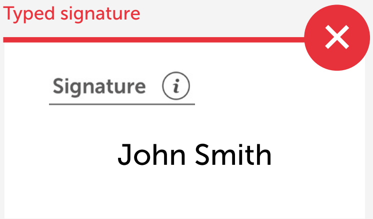

Photograph/screenshot signature

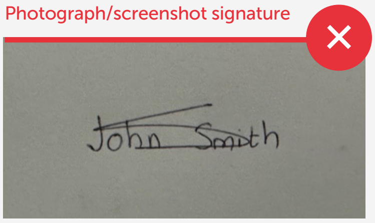

frontiers

Manuscript ID number

1858083

DOI

10.3389/fpubh.2026.1858083

Manuscript title

MLP-CKD: A Clinically Informed Deep Learning Framework for Admission Laboratory-Based Screening and Risk Stratification of Uremia-Associated Advanced Renal Dysfunction

Please tick as appropriate

- [x] Addition of new author(s)
- [ ] Removal of author(s)
- [ ] Addition and removal of author(s)
- [ ] Change in the order of author(s)

Details of author(s) to be added/removed

Complete one table for each author to be added/removed. If you wish to add/remove more than 5 authors, contact the Research Integrity team. Please ensure you provide as much detail as possible in the 'Reason for change' section so we can verify why the change is necessary. This should include details about:

- Why the change is being made.
- Why the author in question was/was not included in the original author list.
- Whether additional work was carried out in response to comments from reviewers and/or editors. If so, please provide details of this work in the 'Individual contributions' section.

If the reason provided is insufficiently detailed or does not address the above points, the Research Integrity team may contact you for additional information. We will be unable to change the author list for your manuscript without sufficient reason being provided.

请为每位需被添加/删除的作者填写单独的申请表格。如果您希望添加/移除5位以上的作者，请您联系相关期刊的研究诚信团队. 请确保在“变更原因”一栏中提供尽可能详细的信息，以便验证作者变更的必要性. 请注意包含以下详细信息：

- 为什么要进行更改.
- 为什么更改的作者在/不在原始作者名单中.
- 对于来自审稿人及编辑的评论是否进行了额外的工作。如果“是”，请在“个人贡献”部分提供该项工作的详细信息.

如果提供的原因不够详细或未解决上述问题，研究诚信团队可能会与您进一步联系已获取更多信息。若无法提供全面且详细的信息，我们将无法进行该稿件作者名单的更改.

frontiers

First name: Yunxia

Surname: Huang

Email: 1484564483@qq.com

Organization name

Department of Nephrology, Pujiang County People's Ho
spital, Jinhua, Zhejiang, China

Addition/removal tick as appropriate

- (●) Addition
- (○) Removal

Individual contributions (as per CRediT Contributor Roles Taxonomy) Only for author additions

Select at least one mandatory contribution:

- (○) Writing – original draft
- (●) Writing – review & editing

Choose any additional optional contribution as required:

- (○) Data curation
- (●) Formal analysis
- (○) Funding acquisition
- (●) Methodology
- (○) Project administration
- (○) Resources
- (○) Supervision
- (○) Validation
- (○) Visualization
- (○) Conceptualization
- (○) Investigation
- (●) Software

Reason for change

The addition of [Yunxia Huang] is requested to accurately acknowledge their substantia
l intellectual contributions during the peer-review revision phase. This author was no
t included in the original submission as their primary involvement commenced after the
 manuscript received reviewer feedback. In response to Reviewer 1’s and the Editor’s
[illegible]

Signature

Only for authors being removed.
Authors being added should sign page 9:

First name: Wen

Surname: Jin

Email: 809337110@qq.com

Organization name

Department of Nephrology, Pujiang County People's Ho
spital, Jinhua, Zhejiang, China

Addition/removal tick as appropriate

- (●) Addition
- (○) Removal

Individual contributions (as per CRediT Contributor Roles Taxonomy) Only for author additions

Select at least one mandatory contribution:

- (○) Writing – original draft
- (●) Writing – review & editing

Choose any additional optional contribution as required:

- (○) Data curation
- (●) Formal analysis
- (○) Funding acquisition
- (●) Methodology
- (○) Project administration
- (○) Resources
- (○) Supervision
- (○) Validation
- (○) Visualization
- (○) Conceptualization
- (○) Investigation
- (○) Software

Reason for change

This authorship addition reflects the substantial contributions of [Wen Jin] during th
e revision process, specifically in addressing the reviewers’ requests for deeper the
oretical interpretation and structural reorganization. They were not listed initially
as their expertise was engaged post-submission to [specify: comprehensively rewrite th
[illegible]

Signature

Only for authors being removed.
Authors being added should sign page 9:

First name: Xiaofeng
Surname: Zhu
Email: 516385742@qq.com

Organization name
Department of Nephrology, Pujiang County People's Hospital, Jinhua, Zhejiang, China

Addition/removal tick as appropriate
- (●) Addition
- ( ) Removal

Individual contributions (as per CRediT Contributor Roles Taxonomy) Only for author additions

Select at least one mandatory contribution:
- ( ) Writing – original draft
- (●) Writing – review & editing

Choose any additional optional contribution as required:
- ( ) Data curation
- (●) Formal analysis
- ( ) Funding acquisition
- (●) Methodology
- ( ) Project administration
- ( ) Resources
- ( ) Supervision
- ( ) Validation
- ( ) Visualization
- ( ) Conceptualization
- ( ) Investigation
- (●) Software

Reason for change
logical transparency]. Their work involved critical revision of the manuscript for important intellectual content, final approval of the revised version, and agreement to be accountable for the accuracy and integrity of the updated sections. All contributions fully satisfy the ICMJE authorship criteria and are documented using the CRediT taxonomy.

Signature
Only for authors being removed.
Authors being added should sign page 9:

First name: Junchen
Surname: Zhao
Email: 448760805@qq.com

Organization name
Department of Nephrology, Pujiang County People's Hospital, Jinhua, Zhejiang, China

Addition/removal tick as appropriate
- (●) Addition
- ( ) Removal

Individual contributions (as per CRediT Contributor Roles Taxonomy) Only for author additions

Select at least one mandatory contribution:
- ( ) Writing – original draft
- (●) Writing – review & editing

Choose any additional optional contribution as required:
- ( ) Data curation
- (●) Formal analysis
- ( ) Funding acquisition
- (●) Methodology
- ( ) Project administration
- ( ) Resources
- ( ) Supervision
- ( ) Validation
- ( ) Visualization
- ( ) Conceptualization
- ( ) Investigation
- ( ) Software

Reason for change
This authorship addition reflects the substantial contributions of [Junchen Zhao] during the revision process, specifically in addressing the reviewers' requests for deeper theoretical interpretation and structural reorganization. They were not listed initially as their expertise was engaged post-submission to [specify: comprehensively rewri [illegible]

Signature
Only for authors being removed.
Authors being added should sign page 9:

First name: Xiaoyi

Surname: Huang

Email: 1347279661@qq.com

Organization name: Department of Nephrology, Pujiang County People's Hospital, Jinhua, Zhejiang, China

Addition/removal tick as appropriate

● Addition ○ Removal

Individual contributions (as per CRediT Contributor Roles Taxonomy) Only for author additions

Select at least one mandatory contribution:

- ○ Writing – original draft
- ● Writing – review & editing

Choose any additional optional contribution as required:

- ○ Data curation
- ● Methodology
- ○ Supervision
- ○ Conceptualization
- ● Formal analysis
- ○ Project administration
- ○ Validation
- ○ Investigation
- ○ Funding acquisition
- ○ Resources
- ● Visualization
- ● Software

Reason for change

This authorship addition reflects the substantial contributions of [Xiaoyi Huang] during the revision process, specifically in addressing the reviewers’ requests for deeper theoretical interpretation and structural reorganization. They were not listed initially as their expertise was engaged post-submission to [specify: comprehensively rewri [illegible]

Signature

Only for authors being removed.
Authors being added should sign page 9:

First name

Surname

Email

Organization name

Addition/removal tick as appropriate

○ Addition ○ Removal

Individual contributions (as per CRediT Contributor Roles Taxonomy) Only for author additions

Select at least one mandatory contribution:

- ○ Writing – original draft
- ○ Writing – review & editing

Choose any additional optional contribution as required:

- ○ Data curation
- ○ Methodology
- ○ Supervision
- ○ Conceptualization
- ○ Formal analysis
- ○ Project administration
- ○ Validation
- ○ Investigation
- ○ Funding acquisition
- ○ Resources
- ○ Visualization
- ○ Software

Reason for change

Signature

Only for authors being removed.
Authors being added should sign page 9:

Provide the author list in the order that you would like it to be published and with the correct spelling.

请按照文章发表时您所希望呈现的作者顺序提供相应的作者名单及作者姓名的正确拼写.

	First name(s)	Surname	Email address	Signature ⓘ	Date
01	Xueliang	Chen	13454922122@163.com	Xueliang Chen	2026.5.27
02	Hao	Yang	haoji@zju.edu.cn	Hao Yang	2026.5.27
03	Yunxia	Huang	1481564483@qq.com	Yunxia Huang	2026.5.27
04	Wen	Jin	809337110@qq.com	Wen Jin	2026.5.27
05	Xiaofeng	Zhu	516385742@qq.com	Xiaofeng Zhu	2026.5.27
06	Junchen	Zhao	448760805@qq.com	Junchen Zhao	2026.5.27
07	Xiaoyi	Huang	1347279661@qq.com	Xiaoyi Huang	2026.5.27
08					
09					
10					
11					
12					
13					
14					
15					
16					
17					
18					
19					
20					
21					
22					
23					
24					
25					

CS 扫描全能王
3亿人都在用的扫描App
